# Comparison of short-term outcomes between direct anterior approach (DAA) and SuperPATH in total hip replacement: a systematic review and network meta-analysis of randomized controlled trials

**DOI:** 10.1186/s13018-021-02315-7

**Published:** 2021-05-20

**Authors:** Nikolai Ramadanov, Simon Bueschges, Kuiliang Liu, Philip Lazaru, Ivan Marintschev

**Affiliations:** 1 Clinic for Emergency Medicine, University Hospital Jena, Friedrich Schiller University, Am Klinikum 1, 07747 Jena, Germany; 2 Faculty of Medicine, Department of Statistics, University of Salamanca, Calle Espejo 2, 37007 Salamanca, Spain; 3 Department for Orthopaedics and Trauma Surgery, Siloah St. Trudpert Hospital, Wilferdinger Str. 67, 75179 Pforzheim, Germany; 4 Center for Surgery, Evangelical Hospital Ludwigsfelde-Teltow, Albert-Schweitzer-Str. 40-44, 14974 Ludwigsfelde, Germany; 5 Department of Trauma, Hand and Reconstructive Surgery, University Hospital Jena, Friedrich Schiller University, Am Klinikum 1, 07747 Jena, Germany

## Abstract

**Background:**

Two minimally invasive approaches showed some advantages in outcomes compared to conventional approaches (CAs)—the direct anterior approach (DAA) and the supercapsular percutaneously assisted approach in THA (SuperPATH). To the best of our knowledge, DAA and SuperPATH have never been compared, neither in clinical studies, nor in a meta-analysis. To conduct a systematic review and network meta-analysis of randomized controlled trials comparing short-term outcomes of DAA and SuperPATH in total hip joint arthroplasty (THA).

**Methods:**

A systematic literature search up to May 2020 was performed to identify randomized controlled trials (RCTs) comparing SuperPATH with CAs and DAA with CAs in THA. We measured surgical, functional, and radiological outcomes. A network meta-analysis, using frequentist methods, was performed to assess treatment effects between DAA and SuperPATH. Information was borrowed from the above-mentioned RCTs, using the CA group as a common comparator.

**Results:**

A total of 16 RCTs involving 1392 patients met the inclusion criteria, three trials with a level I evidence, 13 trials with a level II evidence. The overall network meta-analysis showed that SuperPATH reduced operation time (fixed effect model: MD = 12.8, 95% CI 9.9 to 15.7), incision length (fixed effect model: MD = 4.3, 95% CI 4.0 to 4.5; random effect model: MD = 4.3, 95% CI 0.2 to 8.4), intraoperative blood loss (fixed effect model: MD = 58.6, 95% CI 40.4 to 76.8), and early pain intensity (VAS 1 day postoperatively with a fixed effect model: MD = 0.8, 95% CI 0.4 to 1.2). The two approaches did not differ in acetabular cup positioning angles and in functional outcome.

**Conclusions:**

Our overall findings suggested that the short-term outcomes of THA through SuperPATH were superior to DAA. SuperPATH showed better results in decreasing operation time, incision length, intraoperative blood loss, and early pain intensity. DAA and SuperPATH were equal in functional outcome and acetabular cup positioning.

**Supplementary Information:**

The online version contains supplementary material available at 10.1186/s13018-021-02315-7.

## Introduction

Artificial total hip arthroplasty (THA) was introduced in the twenties of the last century. THA relieves pain, corrects deformities, and improves motor function and quality of life [1]. Several approaches to the hip joint in hip replacement have been described and modified by various authors. They are divided into two main groups: conventional and minimally invasive approaches. Conventional approaches (CAs) are the following: anterior, anterolateral, lateral transgluteal, lateral transtrochanteric, posterior, posterolateral. Minimally invasive approaches are modifications of the CAs with an incision length less than 10 cm [2–4] and a lower tissue traumatization [5–8]. The minimally invasive approaches are divided into two groups: muscle-sparing and mini-incision approaches. However, findings in current literature did not show remarkable benefits in outcomes of minimally invasive approaches compared to CAs in hip replacement [9–15]. Contrary to this general picture, two minimally invasive approaches showed some advantages in outcomes compared to CAs—the direct anterior approach (DAA) and the supercapsular percutaneously assisted approach in THA (SuperPATH). DAA was originally described by the German surgeon Carl Hueter in 1881 [16]. Smith-Petersen popularized DAA with a description in English-speaking literature in 1917 [17]. Judet reported the procedure in 1985 using a traction (fracture) table (TT) [18]. SuperPATH was first described by Chow in 2011 [19]. Table 1 gives a brief comparative overview of the most important DAA and SuperPATH operation points.

**Table 1:** Brief comparative overview of the most important DAA and SuperPATH operation points

	DAA	SuperPATH
**Position**	supine on a regular operating room table or on a TT	lateral decubitus position on a regular operating room table
**Skin incision**	2–4 cm distal and lateral to the ASIS to a point two finger widths anterior to the greater trochanter	from the tip of the greater trochanter in line with the femoral axis
**Deeper preparation**	incision of the fascia over the TFL	incision of the fascia of the gluteus maximus muscle
**Approach to capsule**	Muscle-sparing approach to the capsule through the Hueter interval between the TFL and the rectus femoris	muscle-sparing approach to the capsule through the space between the piriformis posterior and the gluteus minimus and medius muscle anterior
**Further steps**	• capsulotomy with a flap for later repair • placing acetabular retractors in anterosuperior, anteroinferior and posterior location • osteotomy the femoral neck and femoral head removal • acetabular reaming, cup impaction and implantation of the inlay • broaching proximal femur medullary canal with the reamer and implanting the prosthesis stem • reposition and wound closure	• broaching proximal femur medullary canal with the reamer and implanting the prosthesis stem • osteotomy the femoral neck and femoral head removal • capsulotomy • additional distal small incision for the reamer drive shaft and connecting it with the acetabular basket reamer through the main incision • acetabular reaming, cup impaction and implantation of the inlay • reposition and wound closure

*TT* traction table, *ASIS* anterior superior iliac spine, *TFL* musculus tensor fasciae latae

There are numerous systematic reviews and meta-analyses, comparing outcomes between DAA and CAs in hip replacement [20–26]. In general, current literature shows better results for DAA. On the other hand, there are three meta-analyses, comparing the outcomes between SuperPATH and CAs in hip replacement [27–29]. They showed overall better results for SuperPATH.

To the best of our knowledge, DAA and SuperPATH have never been compared, neither in clinical studies, nor in a meta-analysis. The aim of this systematic review and network meta-analysis (NMA) was to compare the short-term outcome of THA through DAA and SuperPATH in treatment of hip diseases and fractures, including only high-quality randomized controlled trials (RCTs).

## Methods

### Reporting guidelines and protocol registration

We followed the Preferred Reporting Items for Systematic Reviews and Meta-Analysis-Protocols (PRISMA-P) guidelines [30]. The review protocol was registered with the International Prospective Register of Systematic Reviews (PROSPERO) on 25 September 2020 and finally approved on 27 October 2020 (CRD42020211298) at http://www.crd.york.ac.uk/PROSPERO/.

### Data sources and search strategies

We searched the following databases and checked citations of screened studies and reviews for relevant manuscripts up to May 2020.

- PubMed
- China National Knowledge Infrastructure (CNKI)
- The Cochrane Library
- Google Scholar
- Clinical trials

We built a BOOLEAN search strategy for studies on DAA and a similar BOOLEAN search strategy for studies on SuperPATH (see appendix for both) and adapted it to the syntax of the used databases. We did not apply restrictions to publication date or language. Results of the searches were exported to a reference management software [31]. A Chinese-speaking reviewer (KL) helped with the search in CNKI.

### Study screening and selection

Two independent reviewers (NR and RK) scanned titles and abstracts to select articles for further consideration. The full text of the selected articles was obtained and scanned again for inclusion by the two reviewers (NR and RK). The decision on inclusion of each study was determined by the consensus between the two reviewers. Cases of disagreement were resolved by discussion and consensus with a third reviewer (KL). Kappa coefficient was used to measure the agreement between the reviewers. A Chinese-speaking reviewer (KL) helped with the study screening and selection by translation of Chinese articles. The entire search and selection process was carried out separately for studies on DAA and studies on SuperPATH, using the same methods.

### Inclusion criteria

Types of studies:

- RCTs

Types of participants:

- Human participants with hip disease or hip fracture

Types of interventions:

- THA through either DAA or SuperPATH compared to CAs

### Exclusion criteria

- No outcome of interest
- Mini-incision approaches
- Employment of a computer navigation system
- Hip replacement with hemiarthroplasty

### Types of outcome measures

## Surgical outcome:

- The operation time (in min) was defined as period of time from the beginning of skin incision to suture. It correlates with the competence of the surgeon as well as risk of infection.
- The incision length (in cm) was measured on graduated scale. It reflects the severity of intraoperative trauma.
- The intraoperative blood loss (in ml) was defined as the total amount of blood from the suction device. It reflects the severity of intraoperative trauma.
- The pain visual analog scale (VAS) is an instrument for measuring pain intensity, providing a range of scores from 0 to 10 [32, 33]. The degree of hip pain was periodically evaluated at certain time intervals after operation.

## Functional outcome:

- The Harris Hip Score (HHS) was developed for assessment of the results of hip surgery [34]. The hip joint function was periodically evaluated at time intervals after operation. The score collects points from the assessment of four aspects: pain, function, degree of deformity, and range of motion of the hip. The higher the added score, the better the results, providing a range of added scores from 0 to 100.

## Radiological outcome

- The acetabular cup anteversion angle and the inclination angle (in degrees) have ideal values for positioning: anteversion angle from 10° to 25° and inclination angle from 40° to 50° [35]. Especially, the ideal acetabular cup anteversion is of great importance, since an angle too large often leads to posterior impingement, resulting in anterior dislocation, and an angle too small leads to posterior dislocation.

### Data extraction and analysis

Data extraction was performed by two reviewers (NR and RK). Cases of disagreement were resolved by discussion and consensus with a third reviewer (KL). We extracted all relevant data into a data extraction form in a standard electronic spreadsheet and the Cochrane software program Review Manager Version 5.3 [36]. We extracted the following data: first author, year of publication, number of patients, patient characteristics, risk of bias, and outcome. A Chinese speaking reviewer (KL) helped with data extraction and analysis by translation of Chinese articles.

### Risk of bias and level of evidence

We examined and checked the selected studies for their risk of bias. We made an assessment using Cochrane’s Risk of Bias 2 (RoB 2) tool [37]. The level of evidence was rated for each study, in accordance with guidelines of the Centre for Evidence-Based Medicine (Oxford, UK) [38].

### Statistical analysis

#### Indirect comparison: network meta-analysis

**Fig. 1:**
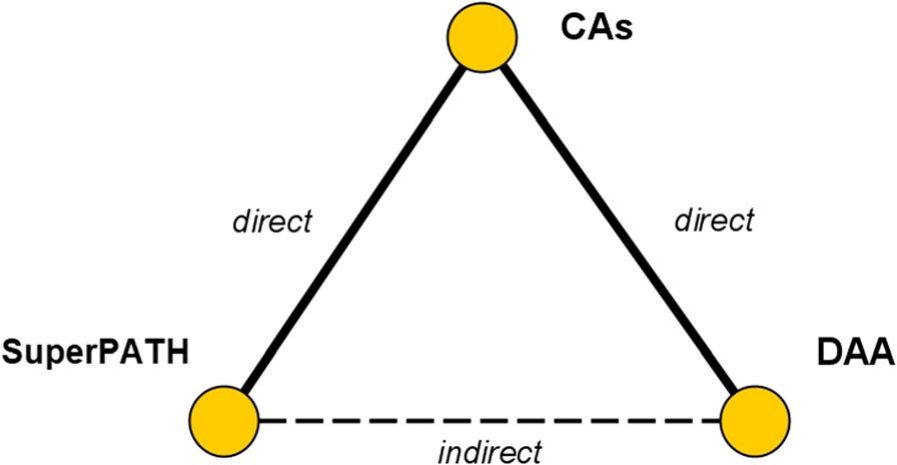
Network plot of the three examined approaches. *DAA* direct anterior approach, *CAs* conventional approaches

A NMA, using frequentist methods [39], was performed to assess treatment effects between DAA and SuperPATH. First, a direct comparison was applied to calculate the results for either DAA or SuperPATH and CAs. Mean differences (MDs) with 95% confidence intervals (CIs) were estimated through fixed and random effects models for all outcomes. Study weighting was performed by inverse variance [40]. Then, information was borrowed from the above-mentioned direct comparisons, using the CA group as a common comparator and reference node within the network. Thereby, effect estimates were obtained in which the difference between the estimations was equivalent to the network estimate. Furthermore, we calculated prediction intervals to estimate where to expect the findings of future NMA on this topic. The network estimates were presented in forest plots. The calculations were done in the R language and environment for statistical computation. From within R, we used the meta [41] and netmeta [42] package. We followed the PRISMA Extension Statement for Reporting of Systematic Reviews Incorporating Network Meta-analyses of Health Care Interventions as basis for the methodology and presentation of the data [43]. All surgical approaches were mapped in a network plot (Fig. 1).

### Assessment of heterogeneity

We assessed clinical and statistical heterogeneity. We did not pool study data that were clinically too diverse. Heterogeneity was assessed using a test on Cochrane’s *Q* statistic, which followed a distribution with k-degrees of freedom (*p* value < 0.10 is indicative of heterogeneity), and a Higgins’ test *I*^2^ (low heterogeneity, < 25%; moderate heterogeneity, 25–75%; and high heterogeneity, > 75%) [44]. The number of degrees of freedom k (*χ*^2^_k_) was equal to the number of studies minus the number of designs. For the distribution of *Q* of a single pooled estimate, *k* equals one, whereas *k* equals two for the network estimate. Results were presented regardless of the detection of heterogeneity in order to maintain the informative value within the forest plots.

## Results

### Study identification and selection

**Fig. 2:**
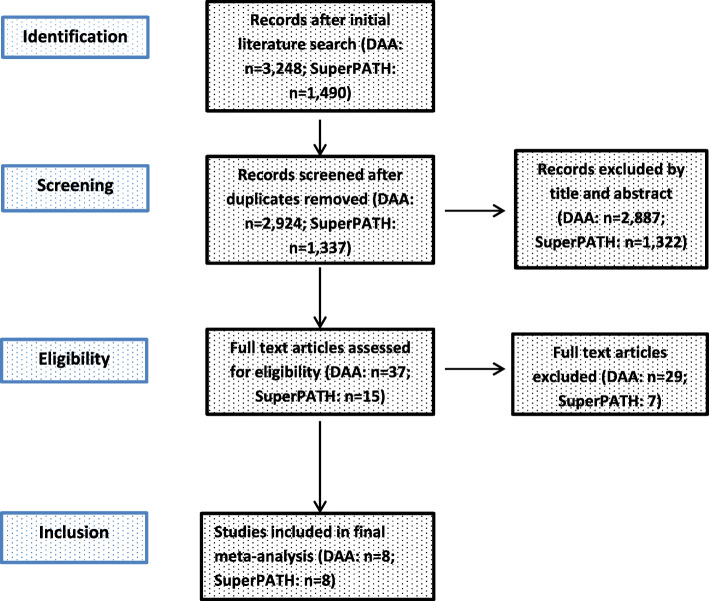
PRISMA flow diagram of the search results and selection according to our inclusion criteria

A description of the study selection process is given in a PRISMA flow diagram (Fig. 2). The PRISMA checklist is given as a Supplemental file.

#### DAA

After removing 324 duplicates, a total of 2924 studies were identified in our initial literature search. Thirty-seven studies were assessed for eligibility after first screening procedure by title and abstract (κ = 0.95) with disagreement between the reviewers concerning 2 studies. Of these studies, 29 were excluded after second screening procedure by full-paper analysis (κ = 1.0), leaving a total of 8 studies on DAA for inclusion in final meta-analysis.

#### SuperPATH

After removing 153 duplicates, a total of 1337 studies were identified in our initial literature search. Fifteen studies were assessed for eligibility after first screening procedure by title and abstract (κ = 1.0) with total agreement by the reviewers. Of these studies, 7 were excluded after second screening procedure by full-paper analysis (κ = 1.0), leaving a total of 8 studies on SuperPATH for inclusion in final meta-analysis.

### Characteristics of the RCTs

The main characteristics of the 16 RCTs on DAA and SuperPATH with overall 1392 included patients are presented in Table 2. The main preoperative diagnoses were osteoarthritis, femoral neck fracture, and avascular necrosis of the femoral head.

**Table 2:** Main characteristics of RCTs included in network meta-analysis

	Sample Size, n		Surgical approach		Mean Age, y (SD or range)		Gender (M/F), n		BMI, kg/m² (SD or range)	
**DAA**										
**Study**	**Pts**	**Hips**	**DAA**	**CAs**	**DAA**	**CAs**	**DAA**	**CAs**	**DAA**	**CAs**
**Barrett 2013** [45]	87	87	43TT	44 pl	61,4±9,2	53,2±7,7	29/14	19/25	30,7±5,4	29,1±5
**D'Arrigo 2009** [46]	169	169	20	149 l	64±8	65±9,8	12/8	81/68	22,7±1,5	28±1,8
**De Anta-Diaz 2016** [47]	99	99	49	50 l	63,5±12,5	64,8±10,1	26/23	26/24	26,9±3,1	26,6±3,9
**Mjaaland 2015** [48]	163	163	83	80 l	67,2±8,6	65,6±8,6	25/58	30/50	3,6±1,9	27,6±3,9
**Nistor 2017** [49]	70	70	35	35 l	67	64	26/9	16/19	27,45±3,76	38,63±3,12
**Reichert 2018** [50]	148	148	77	71 l	63,2±8,2	61,9±7,8	45/32	71/0	28,1±3,7	28,3±3,4
**Rykov 2017** [51]	46	46	23	23 pl	62,8±6,1	60,2±8,1	8/15	11/12	29±5,6	29,3±4,8
**Zhao 2017** [52]	116	120	60	56 pl	64,9±12,1	62,2±14,7	24/36	22/34	24,35±3,1	25,58±2,83
**SuperPATH**										
**Study**	**Pts**	**Hips**	**S**	**CAs**	**S**	**CAs**	**S**	**CAs**	**S**	**CAs**
**Hou 2017** [53]	40	40	20	20 c	54.3 ±13.7	53.8 ±12.9	13/7	12/8	24.5 ±3.6	23.9±4.1
**Meng 2019** [54]	4	8	4	4 pl	51.00 ±4.54		4/0		21.49 (19.60-23.04)	
**Ouyang 2018** [55]	24	24	12	12 pl	54 (45-71)	55 (47-67)	8/4	9/3	23.1 (17.5-26.7)	23.9 (16.9-30.4)
**Ren 2016** [56]	42	42	21	21 c	57.96 ±6.89	58.45±6.25	12/9	13/8	N/A	N/A
**Xie 2017** [57]	92	92	46	46 p	66.6 ±11.88	64.47±12.09	12/34	19/27	23.62 ±1.63	24.06±2.72
**Yan 2017** [58]	154	173	70	103 l	66 (59-75)	65 (56-82)	29/35	42/48	24.5 (17.3-31.1)	23.6 (18-32.3)
**Yuan 2018** [59]	84	84	40	44 pl	74.3 (67-79)	75.7 (69-82)	24/16	21/23	22.73 ±1.71	22.36±1.89
**Zhang 2019** [60]	54	54	27	27 pl	62.41 ±6.44	61.28 ±6.7	10/17	12/15	24.53 ±5.31	23.93 ±4.89

*DAA* direct anterior approach, *S* SuperPATH, *TT* traction table CAs: conventional approaches, *pl* posterolateral approach, *p* posterior approach, *l* lateral approach, *c* conventional approach, *Pts* patients

#### DAA

The 8 studies, comparing DAA with CAs, were published between 2009 and 2018, altogether involving 898 patients (with 902 operated hip joints). Of the included patients, 390 were operated through DAA and 508 through CAs. The sample size of the studies on DAA ranged from 46 to 169 patients. All studies on DAA were published in English language. Of the 8 studies, 3 included conventional THA through posterolateral approach [45, 51, 52], 5 through lateral transgluteal approach [46–50]. 

#### SuperPATH

The 8 studies, comparing SuperPATH with CAs, were published between 2016 and 2019, altogether involving 494 patients (with 517 operated hip joints). Of the included patients, 232 were operated through SuperPATH and 262 through CAs. The sample size of the studies on SuperPATH ranged from 4 to 154 patients. Two studies were published in English language, [54, 57] and 6 studies were published in Chinese with an English abstract [53, 55, 56, 58–60]. Of the 8 studies, 4 included conventional THA through posterolateral approach [54, 55, 59, 60], 1 through posterior approach [57], 1 through lateral transgluteal approach [58]. In 2 studies, the surgical approach was conventional, but not further specified [53, 56].

### Risk of bias and level of evidence

The quality of the included studies was assessed by the Cochrane Collaboration’s tool for risk of bias. Table 3 shows the summarized risk of bias assessment. Three out of 16 studies were blinded RCTs with a level I evidence [48, 52, 54]; the other 13 studies were non-blinded RCTs with a level II evidence [45–47, 49–51, 53, 55–60].

**Table 3:**
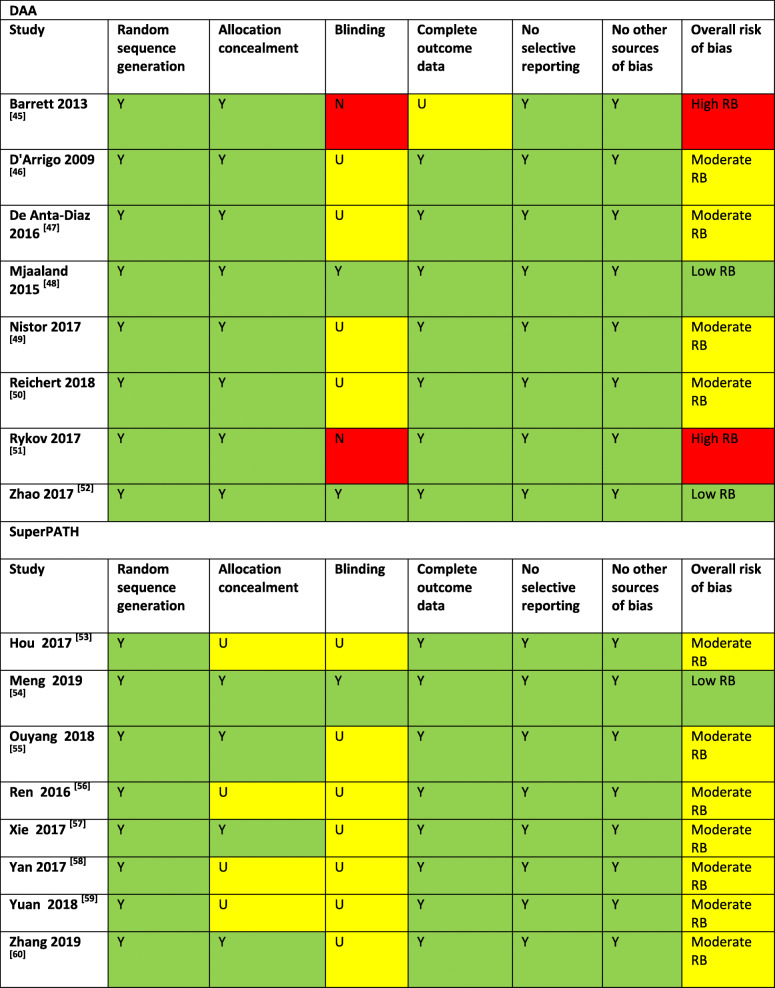
Risk of bias assessment

*DAA* direct anterior approach, *Y* positive result, *U* unclear, *N* negative result, *RB* risk of bias

### Clinical and statistical heterogeneity

**Fig. 3:**
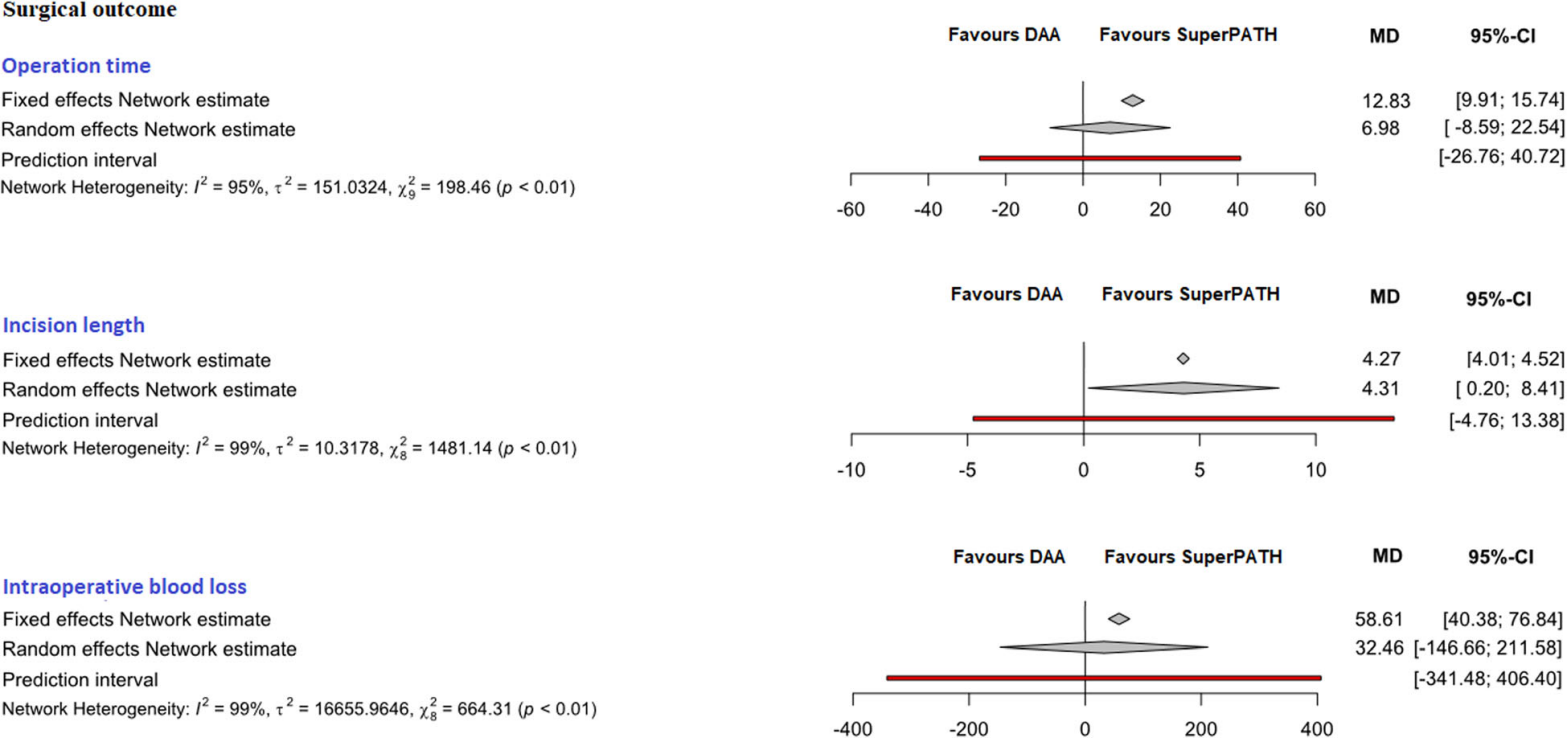
Comparison of the operation time in min., the incision length in cm, the intraoperative blood loss in ml. *DAA* direct anterior approach, *CAs* conventional approaches, *MD* mean difference, *CI* confidence interval

**Fig. 4:**
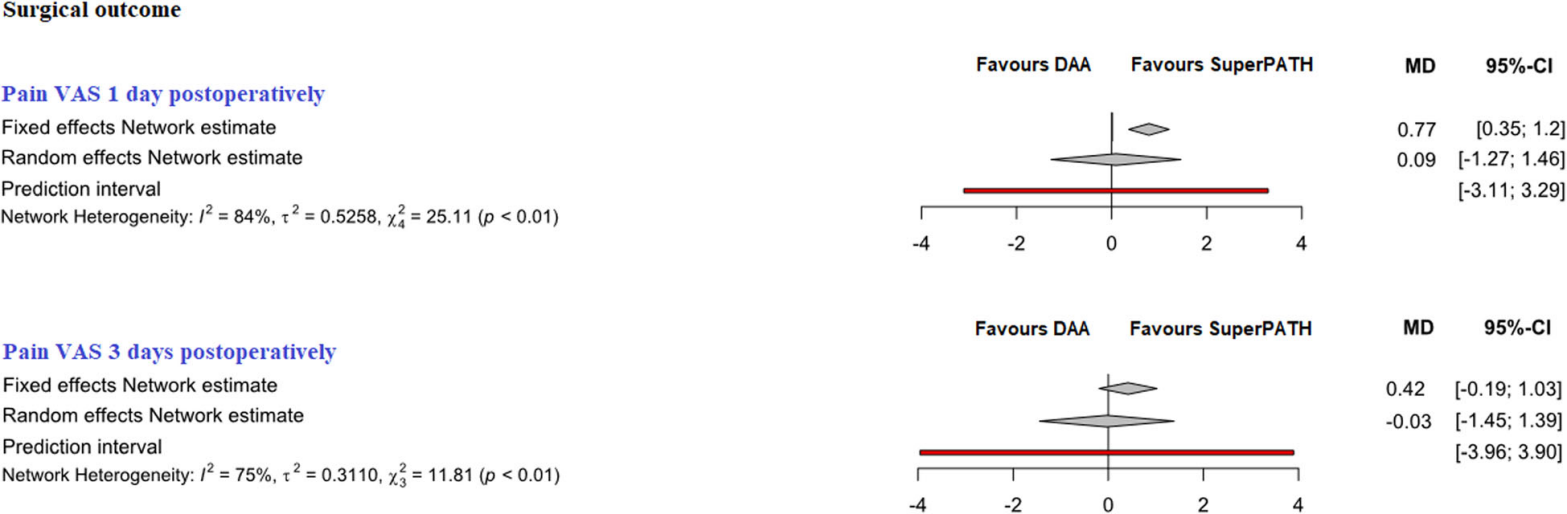
Comparison of the pain VAS 1 day and 3 days postoperatively. *DAA* direct anterior approach, *CAs* conventional approaches, *MD* mean difference, *CI* confidence interval

**Fig. 5:**
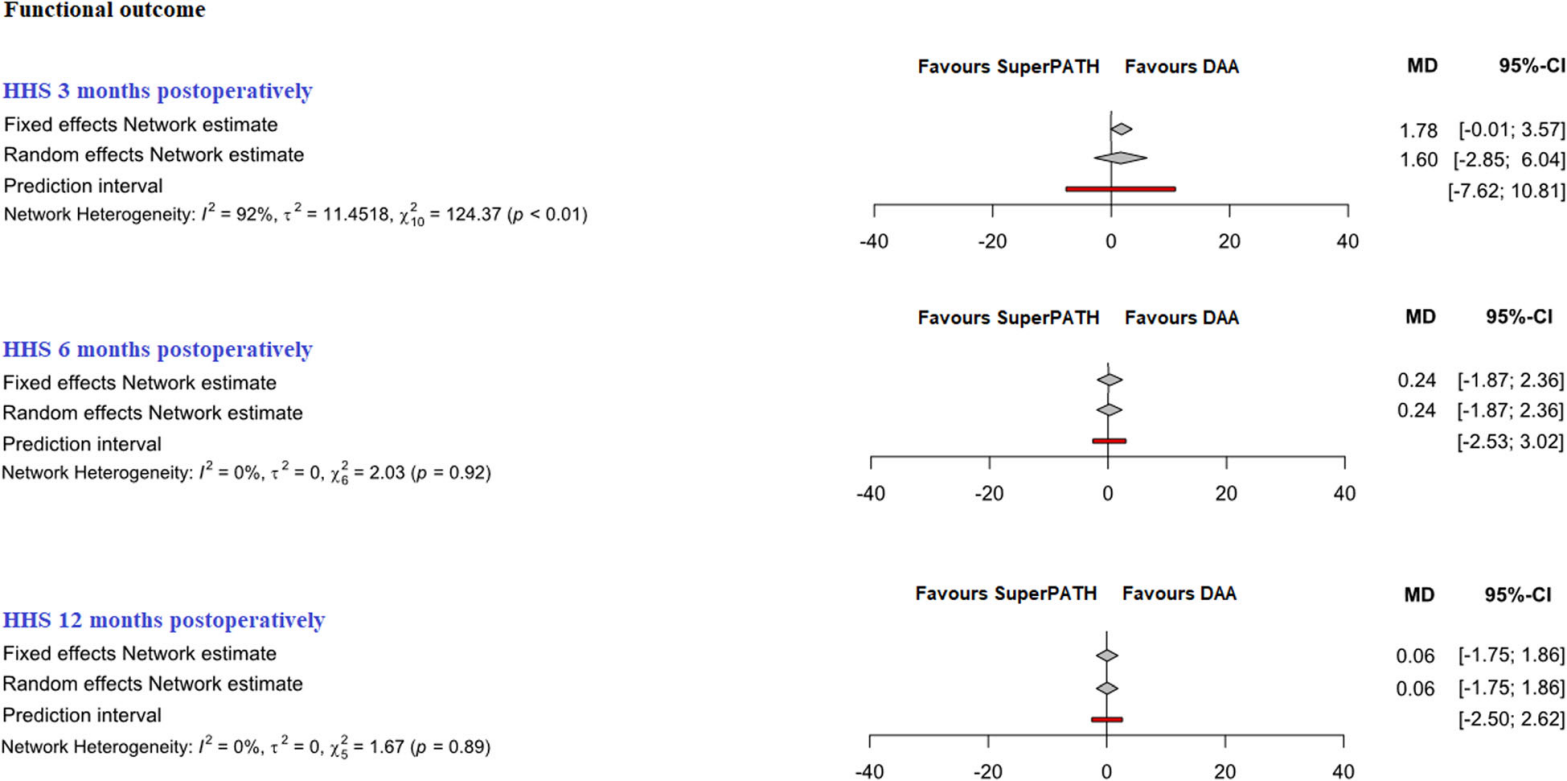
Comparison of the HHS 3, 6, and 12 months postoperatively. *DAA* direct anterior approach, *CAs* conventional approaches, *MD* mean difference, *CI* confidence interval

**Fig. 6:**
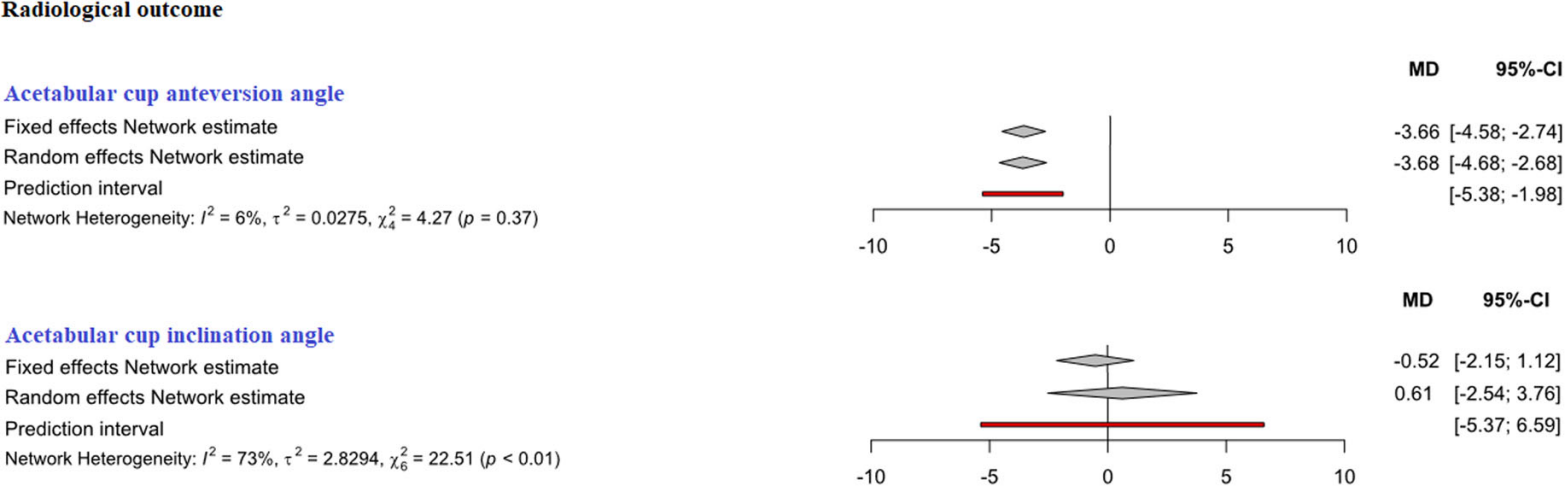
Comparison of the acetabular cup anteversion and inclination angles in degrees. *DAA* direct anterior approach, *CAs* conventional approaches, *MD* mean difference, *CI* confidence interval

No relevant differences were found between the patients in the experimental (either SuperPATH or DAA) and control group (CAs) in clinical characteristics for gender, age and BMI (Table 2). The statistical heterogeneity of all measured outcomes is shown in Figs. 3, 4, 5, and 6.

### Outcomes

#### Surgical outcomes

##### Operation time

In indirect comparison between DAA and SuperPATH, data on 379 patients were pooled from 11 RCTs (*I*^2^ = 95%, *p* < 0.01, Fig. 3, Table 4). The operation time of DAA was 12.8 min longer than the operation time of SuperPATH, using a fixed effect model (MD = 12.8, 95% CI 9.9 to 15.7). There was no difference in operation time, using a random effect model (MD = 7.0, 95% CI − 8.6 to 22.5).

##### Incision length

In indirect comparison between DAA and SuperPATH, data on 371 patients were pooled from 10 RCTs (*I*^2^ = 99%, *p* < 0.01, Fig. 3, Table 4). The incision length of DAA was 4.3 cm longer than the incision length of SuperPATH, using a fixed effect model (MD = 4.3, 95% CI 4.0 to 4.5). The incision length of DAA was 4.3 cm longer than the incision length of SuperPATH, using a random effect model (MD = 4.3, 95% CI 0.2 to 8.4).

**Table 4:** Extracted outcome data from the included studies on DAA and SuperPATH

DAA/Study	Patients (N)	Mean	SD	SuperPATH/Study	Patients (N)	Mean	SD
***Operation time (in min.)***							
Barrett 2013 [45]	43	84.3	12.4	Hou 2017 [53]	20	115	10.1
D’Arrigo 2009 [46]	20	121	23.6	Meng 2019 [54]	2	103.3	12.4
De Anta-Diaz 2016 [47]	49	82.2	15.1	Ouyang 2018 [55]	12	109.6	28.3
Rykov 2017 [51]	23	71	7	Xie 2017 [57]	46	103.6	11.8
Zhao 2017 [52]	60	83.3	6.7	Yan 2017 [58]	64	52	5
				Yuan 2018 [59]	40	57.5	5.7
***Incision length (in cm)***							
Barrett 2013 [45]	43	13.7	0.9	Hou 2017 [53]	20	7.2	0.5
De Anta-Diaz 2016 [47]	49	11.5	0.7	Meng 2019 [54]	2	7.6	1
Nistor 2017 [49]	35	12.2	1.9	Ouyang 2018 [55]	12	10.4	3
Zhao 2017 [52]	60	9.1	0.5	Xie 2017 [57]	46	7.4	1.1
				Yan 2017 [58]	64	5.8	0.6
				Yuan 2018 [59]	40	7.5	1.1
***Intraoperative blood loss (in ml)***							
Barrett 2013 [45]	43	391	206	Hou 2017 [53]	20	315	116
D’Arrigo 2009 [46]	20	1344	710	Meng 2019 [54]	2	1108.5	163.6
Rykov 2017 [51]	23	325.7	99.4	Ouyang 2018 [55]	12	138.3	42.8
Zhao 2017 [52]	60	165.9	42.6	Xie 2017 [57]	46	303.6	106.3
				Yan 2017 [58]	64	349	28
				Yuan 2018 [59]	40	175	11.3
***VAS 1 day postoperatively***							
Barrett 2013 [45]	43	4	1	Hou 2017 [53]	20	3.1	1.3
Mjaaland 2015 [48]	83	2.6	2	Meng 2019 [54]	2	8.3	1
				Ouyang 2018 [55]	12	3.5	0.8
				Yan 2017 [58]	64	4.8	0.6
***VAS 3 days postoperatively***							
Mjaaland 2015 [48]	83	1.6	1.7	Hou 2017 [53]	20	1.5	1.4
				Meng 2019 [54]	2	7	1.4
				Ouyang 2018 [55]	12	2.2	0.7
				Yan 2017 [58]	64	3.1	0.2
***HHS 3 months postoperatively***							
Barrett 2013 [45]	43	91.2	9.7	Meng 2019 [54]	2	72.3	3.9
D’Arrigo 2009 [46]	20	93.1	7.8	Ouyang 2018 [55]	12	82.1	4.8
De Anta-Diaz 2016 [47]	49	94.6	10.2	Ren 2016 [56]	21	86.5	5.1
Reichert 2018 [50]	77	89.8	9.3	Xie 2017 [57]	46	87.6	1.8
Zhao 2017 [52]	60	85.9	17.4	Yan 2017 [58]	64	89.6	2.1
				Yuan 2018 [59]	40	86.5	1.2
				Zhang 2019 [60]	27	83.1	5.5
***HHS 6 months postoperatively***							
Barrett 2013 [45]	43	95.8	7.8	Meng 2019 [54]	2	84.3	6.2
Reichert 2018 [50]	77	90.3	9.8	Ouyang 2018 [55]	12	84.9	5.9
Zhao 2017 [52]	60	92.2	13.3	Yan 2017 [58]	64	93.1	3.7
				Yuan 2018 [59]	40	90	2
				Zhang 2019 [60]	27	88	3.6
***HHS 12 months postoperatively***							
Barrett 2013 [45]	43	97.5	5.7	Meng 2019 [54]	2	92.5	1.7
De Anta-Diaz 2016 [47]	49	96.2	10.1	Ouyang 2018 [55]	12	85.6	6.5
Reichert 2018 [50]	77	92.4	8.6	Xie 2017 [57]	46	92.3	1.6
				Zhang 2019 [60]	27	91.3	3.8
***Acetabular cup anteversion angle (in degrees)***							
Barrett 2013 [45]	43	20.1	5.9	Hou 2017 [53]	20	17.7	1.2
Zhao 2017 [52]	60	17.1	2.1	Meng 2019 [54]	2	15	1.8
				Ouyang 2018 [55]	12	21.9	5.78
				Xie 2017 [57]	46	17.4	1.6
***Acetabular cup inclination angle (in degrees)***							
Barrett 2013 [45]	43	47.1	6.1	Hou 2017 [53]	20	43.8	2.9
Nistor 2017 [49]	35	37	1.9	Meng 2019 [54]	2	38.8	8.2
Reichert 2018 [50]	77	38.6	5.7	Ouyang 2018 [55]	12	37.1	6.5
Zhao 2017 [52]	60	40.3	2.8	Xie 2017 [57]	46	43.6	6.8

*DAA* direct anterior approach, *SD* standard deviation

##### Intraoperative blood loss

In indirect comparison between DAA and SuperPATH, data on 330 patients were pooled from 10 RCTs (*I*^2^ = 99%, *p* < 0.01, Fig. 3, Table 4). The intraoperative blood loss of DAA was 58.6 ml higher than the intraoperative blood loss of SuperPATH, using a fixed effect model (MD = 58.6, 95% CI 40.4 to 76.8). There was no difference in intraoperative blood loss, using a random effect model (MD = 32.5, 95% CI − 146.7 to 211.6).

##### Pain VAS 1 day postoperatively

In indirect comparison between DAA and SuperPATH, data on 224 patients were pooled from 6 RCTs (*I*^2^ = 84%, *p* < 0.01, Fig. 4, Table 4). The pain VAS 1 day postoperatively of DAA was 0.8 points higher than the pain VAS 1 day postoperatively of SuperPATH, using a fixed effect model (MD = 0.8, 95% CI 0.4 to 1.2). There was no difference in pain VAS 1 day postoperatively, using a random effect model (MD = 0.1, 95% CI − 1.3 to 1.5).

##### Pain VAS 3 days postoperatively

In indirect comparison between DAA and SuperPATH, data on 181 patients were pooled from 5 RCTs (*I*^2^ = 75%, *p* < 0.01, Fig. 4, Table 4). There was no difference in pain VAS 3 days postoperatively, using a fixed effect model (MD = 0.4, 95% CI − 0.2 to 1.0). There was no difference in pain VAS 3 days postoperatively, using a random effect model (MD = − 0.1, 95% CI − 1.5 to 1.4).

#### Functional outcome: Harris Hip Score

##### HHS 3 months postoperatively

In indirect comparison between DAA and SuperPATH, data on 461 patients were pooled from 12 RCTs (*I*^2^ = 92%, *p* < 0.01, Fig. 5, Table 4). There was no difference in HHS 3 months postoperatively of DAA, using a fixed effect model (MD = 1.8, 95% CI − 0.1 to 3.6) and a random effect model (MD = 1.6, 95% CI − 2.9 to 6.0).

##### HHS 6 months postoperatively

In indirect comparison between DAA and SuperPATH, data on 325 patients were pooled from 8 RCTs (*I*^2^ = 0%, *p* = 0.92, Fig. 5, Table 4). There was no difference in HHS 6 months postoperatively, using a fixed effect model (MD = 0.2, 95% CI − 1.9 to 2.4) and a random effect model (MD = 0.2, 95% CI − 1.9 to 2.4).

##### HHS 12 months postoperatively

In indirect comparison between DAA and SuperPATH, data on 256 patients were pooled from 7 RCTs (*I*^2^ = 0%, *p* = 0.89, Fig. 5, Table 4). There was no difference in HHS 12 months postoperatively of DAA, using a fixed effect model (MD = 0.1, 95% CI − 1.8 to 1.9) and a random effect model (MD = 0.1, 95% CI − 1.8 to 1.9).

#### Radiological outcome

##### Acetabular cup anteversion angle

In indirect comparison between DAA and SuperPATH, data on 183 patients were pooled from 6 RCTs (*I*^2^ = 6%, *p* = 0.37, Fig. 6, Table 4). The acetabular cup anteversion angle of DAA was 3.7° lower than the acetabular cup anteversion angle of SuperPATH, using a fixed effect model (MD = − 3.7, 95% CI − 4.6 to − 2.7). The in acetabular cup anteversion angle of DAA was 3.7° lower than the acetabular cup anteversion angle of SuperPATH, using a random effect model (MD = − 3.7, 95% CI − 4.7 to − 2.7).

##### Acetabular cup inclination angle

In indirect comparison between DAA and SuperPATH, data on 295 patients were pooled from 8 RCTs (*I*^2^ = 73%, *p* < 0.01, Fig. 6, Table 4). There was no difference in acetabular cup inclination angle, using a fixed effect model (MD = − 0.5, 95% CI − 2.2 to 1.1) and a random effect model (MD = 0.6, 95% CI − 2.5 to 3.8).

## Discussion

### Main and new findings

Sixteen randomized controlled trials with 1392 patients were included in this NMA. The studies on DAA consisted of 898 patients; the studies on SuperPATH consisted of 494 patients. In our NMA, the DAA group consisted of 390 patients, the SuperPATH group of 232 patients, and the CAs group as a common comparator of a total of 770 patients. In general, our NMA indicated that THA through SuperPATH was superior to THA through DAA regarding the investigated outcomes. SuperPATH showed better results on decreasing operation time, incision length, intraoperative blood loss, and early postoperative pain intensity in THA. DAA and SuperPATH were equal in short-term postoperative functional outcome after THA. Furthermore, both approaches showed sufficient results in acetabular cup positioning. Three studies out of 16 were blinded RCTs with a level I evidence [48, 52, 54]; the other 13 studies were non-blinded RCTs with a level II evidence [45–47, 49–51, 53, 55–60].

The value of this NMA comes from the inclusion of high-quality RCTs and the employment of high-quality statistical methods. We calculated the results with both a fixed and a random effect model, offering a higher informative value. Our NMA is the first attempt to systematically and quantitatively review the literature comparing DAA with SuperPATH. To the best of our knowledge, these approaches to the hip joint have never been compared, neither in clinical studies, nor in a meta-analysis.

### DAA vs. SuperPATH

Our indirect comparison between DAA and SuperPATH included 16 RCTs and 1392 patients. The DAA group consisted of 390 patients, the SuperPATH group consisted of 232 patients.There was no difference in operation time, using a random effect model. DAA showed a 12.8 min longer operation time than SuperPATH, using a fixed effect model. This is an important advantage of SuperPATH since prolonged operative times (> 90 min) are associated with increased rates of superficial infections [61]. A 2019 analysis of 89,802 cases of THA by Surace showed that prolonged operation time was associated with perioperative complications [62]. Additionally, the authors suggested an optimal operation time of approximately 80 min with a lower risk of perioperative complications. The mean operation time of the studies included in our NMA ranged from 71 to 121 min for DAA and from 52 to 115 min for SuperPATH. Both approaches are known to have a prolonged learning curve for operating surgeons [63, 64]. SuperPATH may have potential for even shorter operation time, since it is a relatively new approach. In Table 5, our results were compared with the operation time of DAA and SuperPATH from additional studies [65–88, 90–95] . The overall results seem to differ greatly from study to study within the two different approaches, so that a greater influence on the part of the operating surgeon and the clinic can be assumed.

**Table 5:** Comparison of the operation time with additional studies

Study	Mean operation time (in min.)
**DAA**	
Alecci 2011 [65]	89
Berend 2009 [66]	69
Bergin 2011 [67]	78
Brismar 2018 [68]	101
Cheng 2016 [69]	125
Hananouchi 2009 [70]	129.1
Hozack 2008 [71]	57
Ilchmann 2013 [72]	119
Martin 2013 [73]	141
Mayr 2009 [74]	70
Nakata 2009 [75]	104.7
Parvizi 2013 [76]	140
Pogliacomi 2012 [77]	93
Pogliacomi 2012 [78]	111
Rathod 2014 [79]	90
Restreppo 2010 [80]	56.4
Rodriguez 2014 [81]	90
Schweppe 2013 [82]	109
Sebečić 2012 [83]	85
Sendtner 2011 [84]	77
Seng 2009 [85]	73
Spaans 2012 [86]	84
Wayne 2009 [87]	115
Zawadsky 2014 [88]	82.4
**SuperPATH**	
Cai 2017 [89]	89.75
He 2016 [90]	90.1
Huang 2016 [91]	67.4
Más Martínez 2019 [92]	69.5
Li 2017 [93]	80.2
Wang 2020 [94]	108.58
Yun 2017 [95]	119.7

*DAA* direct anterior approach

The mean incision length in our NMA ranged from 9.1 to 13.7 cm for DAA and from 5.8 to 10.4 cm for SuperPATH. DAA had a 4.3 cm longer incision length than SuperPATH, using a fixed and a random effect model. Since both approaches are minimally invasive, they should aim for shorter incision lengths. Nevertheless, literature is inconclusive about the importance of incision length. A 2013 meta-analysis by Xu with 14 RCTs and 1174 patients did not come to a definite overall conclusion whether mini-incision or standard incision THA is superior [96]. Another 2013 meta-analysis by Moskal with 30 studies and 3548 THAs showed that limited incision was superior to standard incision in short-term recovery after THA [97]. Incision length is also dependent on patient weight, height, and gender. Larger and more obese patients as well as women are more likely to receive longer incisions in mini-incision THA [98].

The mean intraoperative blood loss in our NMA ranged from 166 to 1344 ml for DAA and from 138 to 1108 ml for SuperPATH. There was no difference in intraoperative blood loss, using a random effect model. DAA had a 59 ml higher intraoperative blood loss than SuperPATH, using a fixed effect model. The lower blood loss is an important advantage of SuperPATH. In general, literature shows a superiority of mini-incision approaches in reducing blood loss compared to standard approaches [10, 24, 99]. A reason for the higher blood loss of DAA might be a bleeding of branches of the lateral circumflex femoral artery that cross the surgical field when operating through DAA. Sometimes, the ligation of those branches is tedious and time consuming. Other known factors besides approaches to the hip joint influencing blood loss in THA are the utilization of tranexamic acid and intraoperative active warming [100–102]. A 2019 meta-analysis by Qi with 10 RCTs showed that the utilization of intravenous tranexamic acid in patients with hip fracture undergoing hip surgeries reduces blood loss and allogeneic blood transfusion [100]. A 2018 NMA by Fillingham with 34 included studies came to the same conclusion in THA [101].

The mean pain VAS 1 day postoperatively in our NMA ranged from 2.6 to 4 points for DAA and from 3.1 to 8.3 points for SuperPATH. DAA had a 0.8 points higher pain VAS 1 day postoperatively than SuperPATH, using a fixed effect model. There was no difference between DAA and SuperPATH in pain VAS 1 day postoperatively, using a random effect model. Furthermore, there was no difference between DAA and SuperPATH in pain VAS 3 days postoperatively, using a fixed and a random effect model. Postoperative pain is an expected but yet undesirable side effect of all surgical interventions. It has a strong influence on the overall well-being of the patient. The lower pain VAS 1 day postoperatively is an important advantage of SuperPATH. The difference may be due to the innervation of the operation area. Branches of the femoral nerve, the obturator nerve, and cutaneal lateral femoral nerve may contribute to pain sensation, when operating through DAA. In contrast, only branches from Th12 and iliohypogastric nerves contribute to pain sensation, when operating through SuperPATH. Furthermore, the superior-lateral aspect of the capsule may play a greater role than any other region in proprioception and pain perception of the hip joint. However, greater understanding is required in regard to the distribution of capsular innervation according to its anatomical location [103]. A recent 2019 NMA by Liu found that the best way to reduce THA pain 1–2 days postoperatively are the spinal anesthesia and lumbar plexus block [104]. A 2016 NMA by Jiménez-Almonte with 35 RCTs and 2296 patients included found a slight advantage to peripheral nerve blocks compared to local infiltration analgesia and opioid consumption 24 h after THA [105].

The mean HHS 3 months postoperatively in our NMA ranged from 85.9 to 94.6 points for DAA and from 72.3 to 89.6 points for SuperPATH. There was no difference between DAA and SuperPATH in HHS 3, 6, and 12 months postoperatively, using a fixed and a random effect model. Several meta-analyses found that DAA and SuperPATH were superior to CAs in early postoperative functional outcome (HHS 3 months postoperatively) and equal to CAs in subsequent postoperative functional outcomes [23, 24, 26, 27, 29]. Functional outcome is a very important outcome parameter. HHS was developed for the assessment of the results of hip surgery, covering four relevant areas: pain, function, absence of deformity, and range of motion [34].

The mean acetabular cup anteversion angle in our NMA ranged from 17.1 to 20.1° for DAA and from 15.0 to 21.9° for SuperPATH. DAA had a 3.7° lower acetabular cup anteversion angle than SuperPATH, using a fixed and a random effect model. Both approaches stayed within the widely accepted values for acetabular cup positioning: anteversion angle from 10° to 25° [35]. The mean acetabular cup inclination angle in our NMA ranged from 37.0 to 47.1° for DAA and from 37.1 to 43.8° for SuperPATH. There was no difference between DAA and SuperPATH in acetabular cup inclination angle, using a fixed and a random effect model. Both approaches showed a slight tendency toward a flat acetabular cup inclination angle, since the widely accepted values range from 40° to 50° [35].

Intra- and postoperative fractures, especially trochanteric fractures, infections, and hip dislocations, are important complications that seem to show different patterns in certain approaches. Surgical revision rates and leg length discrepancies are also parameters often taken into consideration in comparisons of THA. Nevertheless, postoperative complications could not be compared due to lack of consistent data in the RCTs included.

### Limitations

We found the following limitations to our NMA: First, the long-term outcomes in THA were not considered. Second, due to insufficient data, important outcome parameters such as hospitalization time, postoperative drainage volume, and postoperative complications could not be considered. Third, this NMA did not consider the possible influence of the surgeon operating skills, the utilization of tranexamic acid and anticoagulants, bone cement, or the types of implants for hip replacement. Fourth, part of the studies did not give any information what exact hip pathology was treated with THA. Fifth, since the SuperPATH approach is a 2-incision approach, it remains unclear whether the included RCTs reported the added incision length or the length of the larger incision, ignoring the smaller additional incision. Sixth, the direct comparison probably offers a statistically higher quality meta-analysis. Since there are no RCTs comparing DAA with SuperPATH, at this point we cannot carry out and offer anything other than an indirect comparison. Lastly, in some cases of the outcomes investigated, the heterogeneity of the included RCTs was high.

## Conclusion

Our overall findings suggested that the short-term outcomes of THA through SuperPATH were superior to DAA. SuperPATH showed better results in decreasing operation time, incision length, intraoperative blood loss, and early pain intensity, using a fixed effect model. SuperPATH showed equal results to DAA in operation time, intraoperative blood loss, and early pain intensity; it showed better results than DAA in incision length, using a random effect model. DAA and SuperPATH were equal in functional outcome and acetabular cup positioning.

### Supplementary Information

**Additional file 1.**

## Data Availability

The data are available from the corresponding author upon reasonable request.

## Appendix

SuperPATH vs. CAs

I. Search strategy PubMed:

((SuperPATH) OR (Supercapsular Percutaneously-Assisted Total Hip)) ti,ab.

II. Search strategy CNKI:

(SuperPATH) OR (Supercapsular Percutaneously-Assisted Total Hip) in Title

III. Search strategy Cochrane Library:

((SuperPATH) OR (Supercapsular Percutaneously-Assisted Total Hip)) in Title Abstract Keyword

IV. Search Strategy Google Scholar:

(SuperPATH) OR (Supercapsular Percutaneously-Assisted Total Hip)

V. Search strategy Clinical Trials:

(SuperPATH) OR (Supercapsular Percutaneously-Assisted Total Hip)

DAA vs. CAs

I. Search strategy PubMed:

(((AMIS) OR anterior minimally invasive surgery) OR direct anterior Approach) AND ((total hip replacement) OR total hip arthroplasty)

II. Search strategy CNKI:

(直接前路入路)

III. Search strategy Cochrane Library:

(((AMIS) OR anterior minimally invasive surgery) OR direct anterior Approach) AND ((total hip replacement) OR total hip arthroplasty)

IV. Search strategy Google Scholar:

AMIS direct anterior approach versus conventional approach total hip replacement randomized controlled trial

V. Search strategy Clinical Trials:

(((AMIS) OR anterior minimally invasive surgery) OR direct anterior Approach) AND ((total hip replacement) OR total hip arthroplasty)

## Abbreviations

CNKI: China National Knowledge Infrastructure
CI: Confidence interval
DAA: Direct anterior approach
HHS: Harris hip score
MD: Mean difference
NMA: Network meta-analysis
RCT: Randomized controlled trial
SuperPATH: Supercapsular percutaneously assisted approach in total hip arthroplasty
THA: Total hip arthroplasty
TT: Traction table
VAS: Visual analog scale
